# Genome-wide identification and analysis of the *GGCT* gene family in wheat

**DOI:** 10.1186/s12864-023-09934-w

**Published:** 2024-01-04

**Authors:** Long Zhang, Wanting Sun, Weidong Gao, Yanyan Zhang, Peipei Zhang, Yuan Liu, Tao Chen, Delong Yang

**Affiliations:** 1 State Key Laboratory of Aridland Crop Science, Lanzhou, Gansu 730070 China; 2 College of Life Science and Technology, Gansu Agricultural University, Lanzhou, Gansu 730070 China

**Keywords:** Wheat, *GGCT* gene family, Genome-wide identification, Expression pattern, Haplotype analysis

## Abstract

**Background:**

γ-glutamylcyclotransferase (GGCT), an enzyme to maintain glutathione homeostasis, plays a vital role in the response to plant growth and development as well as the adaptation to various stresses. Although the *GGCT* gene family analysis has been conducted in Arabidopsis and rice, the family genes have not yet been well identified and analyzed at the genome-wide level in wheat (*Triticum aestivum* L.).

**Results:**

In the present study, 20 *TaGGCT* genes were identified in the wheat genome and widely distributed on chromosomes 2A, 2B, 2D, 3A, 4A, 5A, 5B, 5D, 6A, 6B, 6D, 7A, 7B, and 7D. Phylogenetic and structural analyses showed that these *TaGGCT* genes could be classified into three subfamilies: ChaC, GGGACT, and GGCT-PS. They exhibited similar motif compositions and distribution patterns in the same subgroup. Gene duplication analysis suggested that the expansion of *TaGGCT* family genes was facilitated by segmental duplications and tandem repeats in the wheat evolutionary events. Identification of diverse *cis*-acting response elements in *TaGGCT* promoters indicated their potential fundamental roles in response to plant development and abiotic stresses. The analysis of transcriptome data combined with RT-qPCR results revealed that the *TaGGCTs* genes exhibited ubiquitous expression across plant organs, with highly expressed in roots, stems, and developing grains. Most *TaGGCT* genes were up-regulated after 6 h under 20% PEG6000 and ABA treatments. Association analysis revealed that two haplotypes of *TaGGCT20* gene displayed significantly different Thousand-kernel weight (TKW), Kernel length (KL), and Kernel width (KW) in wheat. The geographical and annual distribution of the two haplotypes of *TaGGCT20* gene further revealed that the frequency of the favorable haplotype *TaGGCT20-Hap-I* was positively selected in the historical breeding process of wheat.

**Conclusion:**

This study investigated the genome-wide identification, structure, evolution, and expression analysis of *TaGGCT* genes in wheat. The motifs of TaGGCTs were highly conserved throughout the evolutionary history of wheat. Most *TaGGCT* genes were highly expressed in roots, stems, and developing grains, and involved in the response to drought stresses. Two haplotypes were developed in the *TaGGCT20* gene, where *TaGGCT20-Hap-I*, as a favorable haplotype, was significantly associated with higher TKW, KL, and KW in wheat, suggesting that the haplotype is used as a function marker for the selection in grain yield in wheat breeding.

**Supplementary Information:**

The online version contains supplementary material available at 10.1186/s12864-023-09934-w.

## Introduction

Glutathione (GSH) [1], a natural tripeptide composed of γ-glutamy, cysteinyl, and glycine, is involved in the intracellular γ-glutamyl cycle, the transportation of amino acids and other nutrients metabolism activity [2]. GSH metabolism can promote the formation of disulfides, thioethers and scavenge free radicals in living organisms [3]. It is one of the most effective scavengers of peroxides produced during intracellular metabolism and oxidative stress in plants [3, 4]. GGCT is an essential enzyme involved in the catabolism of the γ-glutamyl cycle, which plays an important role in the degradation of GSH and the maintenance of GSH homeostasis [4]. GSH has been observed to convert to 5-oxoproline (5OP) by a combination of GGCT and 5-oxoprolinase in the cytoplasm. Subsequently, 5OP was converted to glutamate and played a vital role in plant homeostasis [5].

GGCT, an enzyme with GSH degradation activity, has been discovered and characterized in mammals [4]. Subsequently, three homologs namely GGCT2;1, GGCT2;2, and GGCT2;3, have been identified in Arabidopsis. In vitro experiments demonstrated that all three homologs degrade GSH at physiological concentrations. Furthermore, these homologs have been shown to successfully complement the function of GSH degradation-defective yeast mutant [2, 6]. GGCT was involved in a variety of biological processes in *Arabidopsis thaliana*, such as pollen tube growth [7], heavy metal stress response [8], salinity stress response [9, 10], and starvation for the essential macronutrient sulfur (S) [11, 12]. GGCT2, as a protein with a cation transport regulatory structural domain, has been reported to protect plants from heavy metal toxicity by recycling glutamic and ensuring the dynamic homeostasis of GSH in response to abiotic stresses [2]. In addition, the promoter of *GGCT2;1* contains the SURE *cis*-element (SUlfur Response Element), which has been found in the promoters of many genes that were transcriptionally up-regulated by S-starvation [10]. The up-regulated expression of *GGCT2;1* in response to sulfur deficiency play a critical role in the regulation of root architecture by controlling GSH homeostasis [10]. The *GGCT* genes have also been preliminarily studied in rice [13]. *OsGGCT-1* and *OsGGCT-2* expression are significantly up-regulated under drought and salt stress, suggesting that they involves in the response to salt and drought tolerance in rice [14].

As one of the most important staple food crops in the world, wheat (*Triticum aestivum* L.) is one of important food crops worldwide, accounting for one-fifth of calories consumed [15]. However, many biological function genes have not yet been well studied in wheat due to the heterologous hybridization and large genome size (~ 17 GB) [16]. Although the functions and regulatory mechanisms of *GGCT* genes have been studied in Arabidopsis and rice [2, 13, 17], the knowledge of their distribution, evolution, and function is still limited in wheat. In the present study, we systematically performed a genome-wide analysis of the wheat *GGCT* gene family to investigate their gene structures, phylogenetic relationship, genomic syntenic analysis, and *cis*-acting element analysis. The tissue-specific and expression patterns under different stress treatments of these genes were also examined using the transcriptomic data and real-time quantitative PCR (RT-qPCR). In addition, we used wheat resequencing data from the WheatUnion database and grain trait data acquired from published work [18–22] to perform association analysis with *TaGGCT*20 gene. The findings provided a good insight into the evolution and function of *GGCT* family genes.

## Results

### Identification of *GGCT* genes in wheat

In this study, 20 members of the *TaGGCT* family genes were eventually identified and named *TaGGCT1* to *TaGGCT20* (Table S1). The family members have been confirmed to possess the conserved GGCT domain based on the results obtained from the NCBI-CDD (https://www.ncbi.nlm.nih.gov/Structure/bwrpsb/bwrpsb.cgi), Inter Pro (http://www.ebi.ac.uk/interpro/scan.html) and SMART (http://smart.embl-heidelberg.de/) databases [23–25]. These genes were unevenly distributed on chromosomes 2A, 2B, 2D, 3A, 4A, 5A, 5B, 5D, 6A, 6B, 6D, 7A, 7B and 7D. The length of the coding regions of *GGCT* genes ranged from 300 (*TaGGCT15*) to 2278 bp (*TaGGCT6*). The relative molecular weights (Mw) of proteins encoded by *TaGGCT* genes ranged from 11.34 (TaGGCT15) to 24.63 aa (TaGGCT19), and the isoelectric points (pI) ranging from 4.5 (TaGGCT5) to 9.13 (TaGGCT15). The predicted subcellular localization showed that most TaGGCT proteins were localized in the cytoplasm (Table S1).

### Phylogenetic, gene structure and conserved motif analysis of *GGCT* genes

**Fig. 1:**
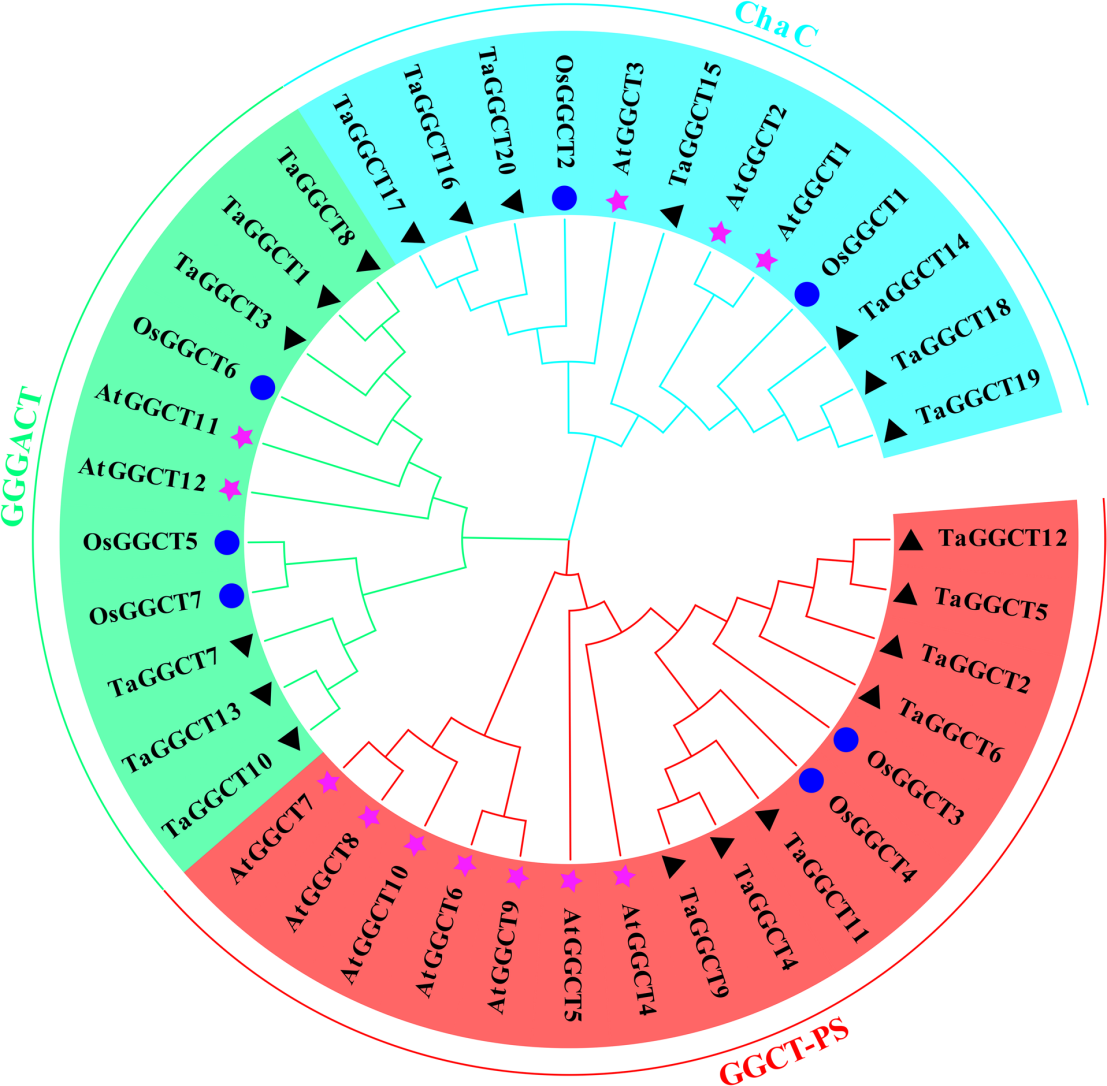
Phylogenetic tree analysis of GGCTs. Note: Different colour blocks indicate different subclasses of the *TaGGCT* gene family

To confirm the genetic evolution relation of all putative TaGGCTs, a phylogenetic tree of the TaGGCTs in wheat was constructed. The results showed that GGCTs proteins were classified into three groups: ChaC, GGGACT, and GGCT-PS (Fig. 1). Interestingly, some genes of GGCT-PS subfamily (*AtGGCT6/10*) did not find their homologous in wheat, suggesting that they could be lost during the wheat evolution process.

**Fig. 2:**
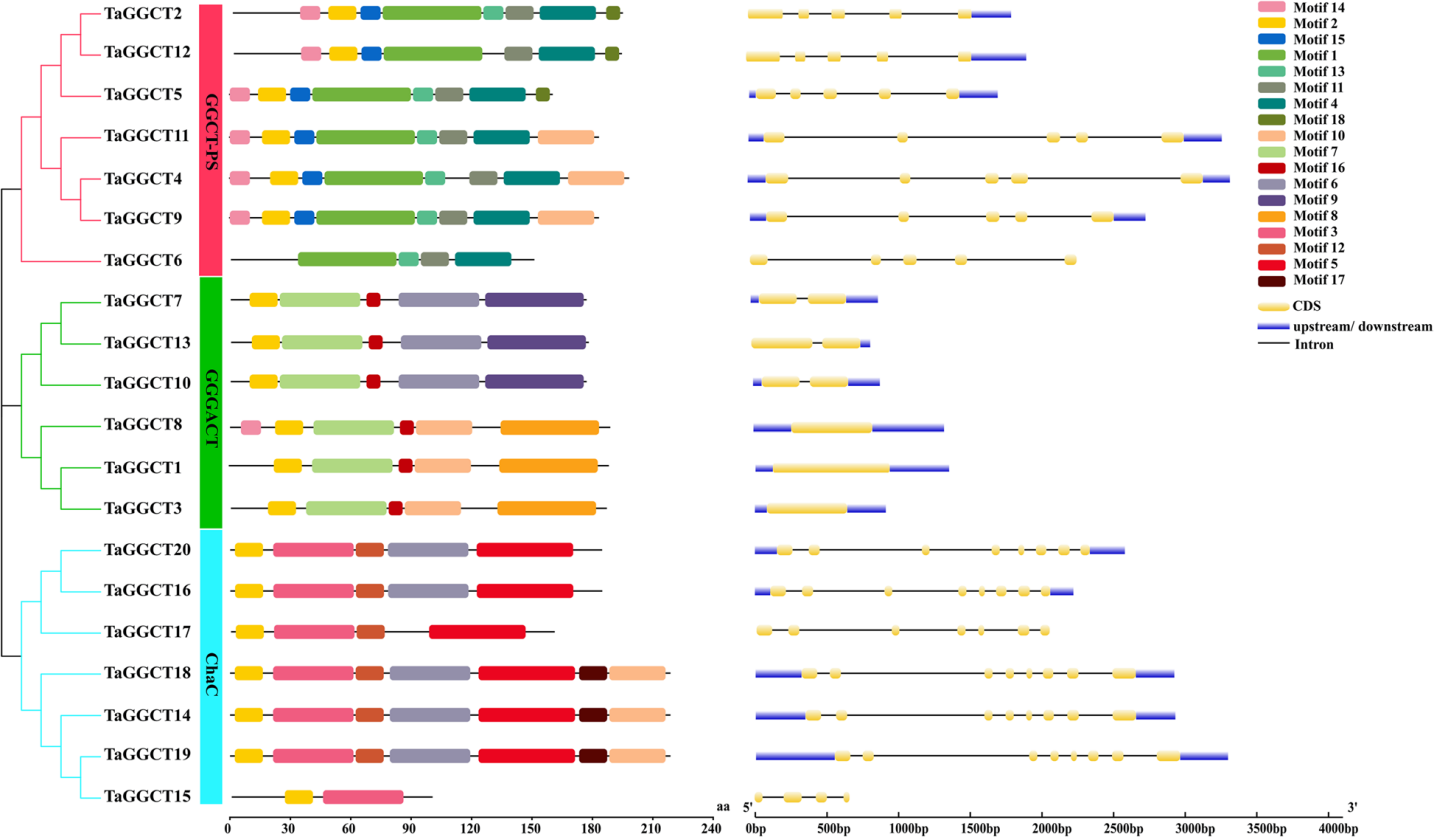
Phylogenetic relationships, gene structure and conserved motifs of *TaGGCT* genes in wheat. Note: Structure of the TaGGCT genes, blue boxes represent UTRs, yellow boxes represent exons, and black lines represent introns

The conserved motifs of wheat TaGGCT proteins were identified by the MEME database, thereby providing clarification on the structural diversity of these proteins. A total of 18 conserved motifs were identified (Fig. 2). The NCBI-CDD database revealed that motif 2 was a conserved structural domain of GGCT_like superfamily, which was relatively conserved among all members of the GGCTs. Proteins in the same TaGGCT subfamily had similar motif compositions and distribution patterns. Of these, the motif 1, motif 4, motif 11, and motif 13 were only found in the GGCT-PS subfamily. The motif 6 and motif 7 were observed in the GGGACT subfamily and the motif 3 specifically existed in the ChaC subfamily. This suggested that they could possess evolutionarily conserved features with possibly similar functions (Fig. S1).

The structures of *TaGGCT* genes were analyzed and visualized using the GSDS software and the results showed that most members within the same subfamily generally shared similar exon/intron structure (Fig. 2). For example, all the GGCT-PS subfamily genes contained five exons and four introns. The ChaC subfamily contained eight exons and seven introns, except for *TaGGCT15*, which contained 4 exons and 3 introns. In the GGCT-PS subfamily, *TaGGCT7*, *TaGGCT10*, and *TaGGCT13* only contained one intron, while *TaGGCT1*, *TaGGCT3*, and *TaGGCT8* did not have any introns. This suggested that *TaGGCT* genes were highly conserved in the duration of the wheat evolution.

### Chromosomal localization and gene duplication in the wheat *TaGGCTs*

**Fig. 3:**
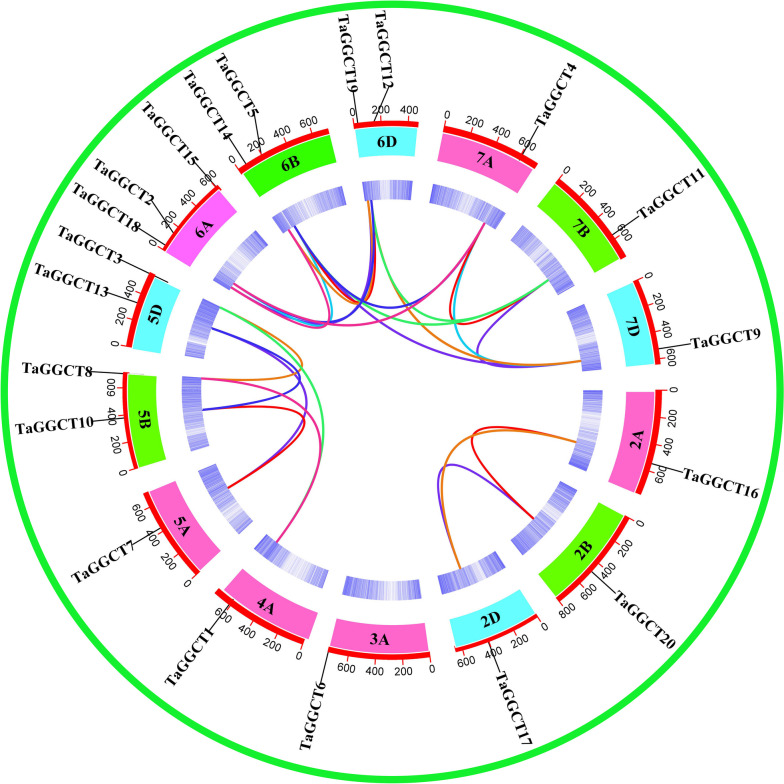
Collinearity analysis of *TaGGCT* genes. Note: Color lines indicate fragment replication, the innermost circle in the red circle is the wheat genome density heat map, and the outermost circle is the name and size of the wheat chromosome

The results of chromosomal location showed that the *TaGGCT* genes were unevenly distributed on chromosomes 2A, 2B, 2D, 3A, 4A, 5A, 5B, 5D, 6A, 6B, 6D, 7A, 7B, and 7D (Fig. S2). To gain more insight into the expansion and evolution of *TaGGCT* family genes in wheat, a syntenic analysis of the family genes was performed using the MCScanX program. A total of 24 co-linear *GGCT* gene pairs were identified, including two pairs on chromosome 4A, three pairs on 2A/2B and 7A/7B, four pairs on 5A/5B, and 12 pairs on 6A/6B/6D (Fig. 3, Table S2). Tandem duplication was not identified in *TaGGCT* family genes. Based on the Ka/Ks ratio of duplicated genes as a marker of selection pressure, the Ka/Ks (< 1) showed that *TaGGCT* genes were driven by purifying selection pressure after duplication events (Table S2). In addition, syntenic analysis of *TaGGCT* genes among rice, wheat and maize revealed that 17 pairs of *TaGGCT* genes were homologous with *OsGGCT* genes in rice and 13 pairs were homologous with *ZmGGCT* genes in maize (Fig. S3). This suggested that *GGCT* genes could be closely related in different species, along with similar biological functions.

### Analysis of *cis*-acting elements of the wheat *TaGGCT* genes

*Cis*-acting elements in gene promoters are crucial regions for initiating transcription at transcription factor-binding sites, thus revealing the differences in transcript expression and biological function of different genes. The promoter sequence in the 2000 bp upstream of the transcriptional start site of *TaGGCT* family genes were analyzed by the PlantCARE software. Except for the basic *cis*-acting elements (CAAT-box and TATA-box), 29 *cis*-acting elements were screened and classified into four categories, including six for plant growth and development elements, six for phytohormone response elements, five for light response elements, and 12 for stress regulation elements (Fig. S4, Table S3). The presence of different numbers and types of *cis*-acting elements in *TaGGCTs* promoters indicates that these genes might be involved in different regulatory mechanisms.

### Organ-specific expression profiles of the wheat *TaGGCT* genes

**Fig. 4:**
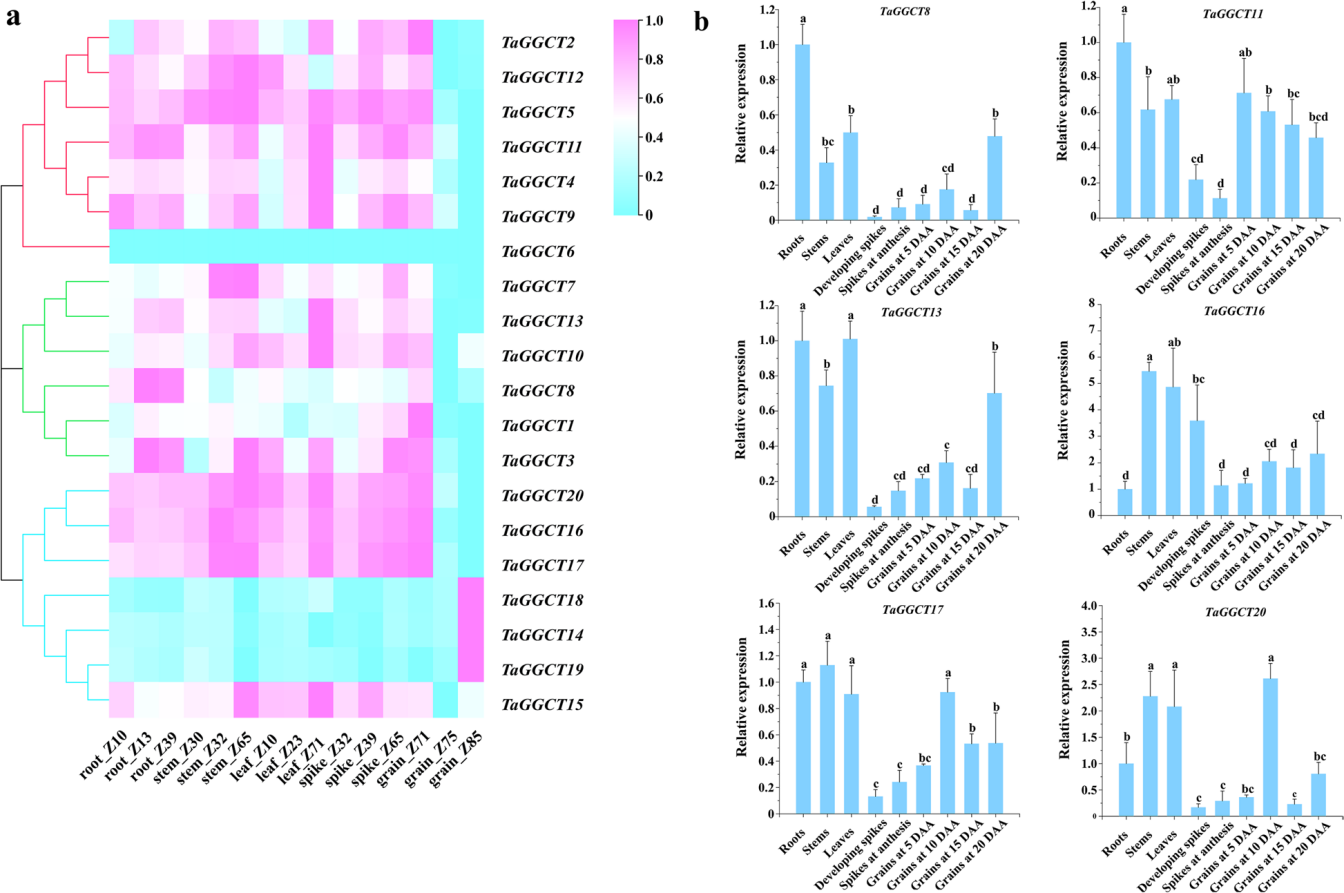
Expression analysis of the *TaGGCT* genes. **a** Expression heatmap of *TaGGCT* genes in different growth stages and different tissues of Chinese Spring. **b** RT-qPCR analysis of *TaGGCTs* gene in different tissues and developmental periods in wheat. Note: Means and standard deviations (SDs) were calculated from three biological and three technical replicates. The error bars indicate the standard deviation. Tubulin was used as a reference gene. Z10: One-leaf stage; Z13: Three-leaf stage; Z23: Early tiller stage; Z30: Booting stage; Z32: Early jointing stage; Z39: Late jointing stage; Z65: Mid-flowering stage; Z71: 2 d after flowering; Z75: 10 d after flowering; Z85: 30d after flowering

The specific expression of genes in different plant organs and developmental stages provides important information about their biological function. Therefore, the expression level of wheat *GGCT* genes in roots, stems, leaves, spikes, and grains at different developmental stages were analyzed using wheat transcriptome data from the Wheat Multi-omics database. The results showed that most *TaGGCT* genes were expressed as a broad-spectrum pattern in different organs, while three genes in subfamily ChaC, *TaGGCT14*, *TaGGCT18*, and *TaGGCT19*, only specifically expressed in nearly mature grains (Fig. 4a). This indicated these three genes could be involved in the regulation of grain development via some unknown pathway. To further verify the expression pattern of *TaGGCT* genes, RT-qPCR was applied to detect the expression level of six *TaGGCT* genes in different organs, including root, stem, leaf, developing spikes, spikes at anthesis, and developing grains at 5, 10, 15, and 20 Days after anthesis (DAA), respectively. The RT-qPCR results showed that most of genes were expressed at a higher level in the vegetative organs than the reproductive organs (Fig. 4b). Of these, the expression levels of *TaGGCT11*, *TaGGCT13*, and *TaGGCT17* were significantly higher in roots, stems and leaves than developing spikes, spikes at anthesis and grain at 5–20 DAA. *TaGGCT8* showed the highest expression level in roots. Interestingly, *TaGGCT20* and *TaGGCT13* were highly expressed in developing grains at 10 DAA and 20 DAA, respectively. Thus, *TaGGCT* genes might serve diverse roles in the growth of vegetative organs, as well as the development of spike and grain in wheat at different expression patterns.

### Stress-induced expression profiles of the wheat *TaGGCT* genes

**Fig. 5:**
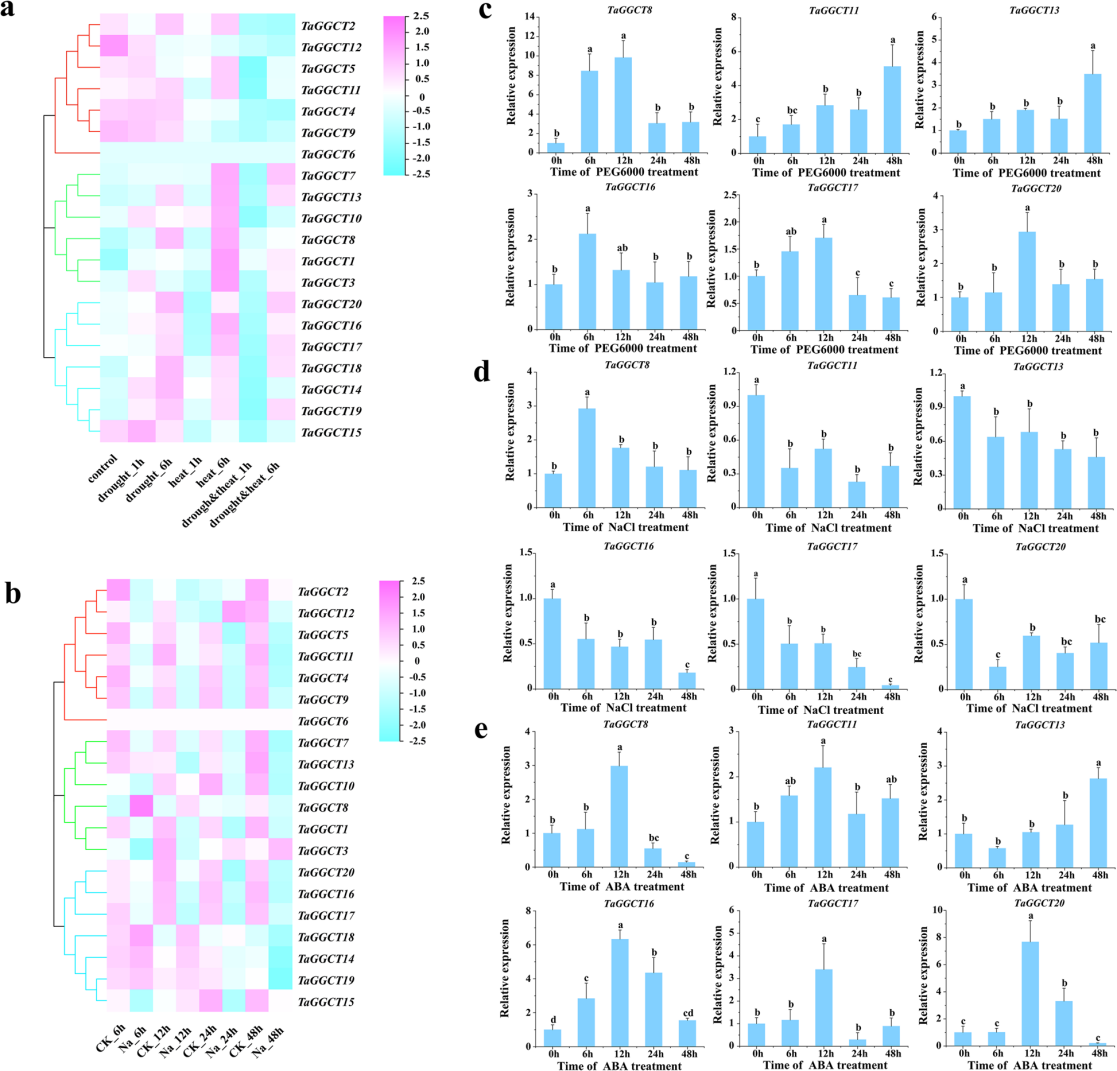
Expression analysis of *TaGGCT* genes in wheat under different treatments. **a** Expression heatmap of *TaGGCT* genes under drought and heat stress treatments. **b** RT-qPCR analysis of *TaGGCT* genes under PEG6000 treatment. **c** Expression heatmap of *TaGGCT* genes under salt stress treatment. **d** RT-qPCR analysis of *TaGGCT* genes under NaCl treatment. **e** RT-qPCR analysis of *TaGGCT* genes under ABA treatment. Note: Each bar value is the average value ± standard deviation based on three biological replicates. TaActin1 was used as a reference gene

Since multiple abiotic stress-related elements were identified in the promoter of *TaGGCT* genes, the expression pattern of *TaGGCT* genes under different abiotic stress treatments were analyzed using transcript data from the wheat multi-omics database (Fig. S4, Table S3). The results showed that *TaGGCT* genes in the same subclade with a similar expression pattern (Fig. 5a). For instance, the expression levels of *TaGGCT14*, *TaGGCT16*, *TaGGCT17*, *TaGGCT18*, *TaGGCT19*, and *TaGGCT20* in subclade ChaC were up-regulated after 6 h under drought-stressed treatment, except for *TaGGCT15*, which was up-regulated after 1 h under the treatment. Among GGGACT subfamily, *TaGGCT3* and *TaGGCT10* were up-regulated after 1 h under drought-stressed treatment, while *TaGGCT8* and *TaGGCT13* were up-regulated after 6 h under the treatment. In subclade GGCT-PS, the expression of *TaGGCT4*, *TaGGCT5*, and *TaGGCT9* was insensitive to drought-stressed treatment, while *TaGGCT12* was significantly down-regulated under drought-stressed treatment. Under NaCl treatment, the expression of most genes was significantly down-regulated in the GGGACT and GGCT-PS subfamilies, while *TaGGCT8* and *TaGGCT12* were highly expressed after 6 and 24 h under NaCl treatment, respectively. In the ChaC subfamily, the expression levels of *TaGGCT15*, *TaGGCT16*, *TaGGCT17*, and *TaGGCT20* were consistently decreased from 0 h ~ 48 h after NaCl treatment, whereas *TaGGCT14*, *TaGGCT18*, and *TaGGCT19* were up-regulated after 6 h under the treatment (Fig. 5b). Therefore, it was speculated that different subfamilies might have functional differentiation to response to certain abiotic stress.

To further verify the function of *TaGGCT* genes in response to abiotic stress, the expression level of six *TaGGCT* genes significantly induced under different abiotic stress treatment were investigated by RT-qPCR under PEG (20% PEG6000), NaCl (200 mM) and ABA (100 µM) treatments. Under PEG treatment, the expression levels of six genes were significantly up-regulated (Fig. 5c). In particular, *TaGGCT8* was rapidly up-regulated after 6 h under drought-stressed treatment and peaked at 12 h, where the expression level was 10-fold higher than that of 0 h. Under NaCl treatment, the expression of *TaGGCT* genes, including *TaGGCT11*, *TaGGCT13*, *TaGGCT16*, *TaGGCT17*, and *TaGGCT20* were significantly down-regulated, while *TaGGCT8* was significantly induced by NaCl treatment after 6 h (Fig. 5d).

ABA pathway is generally involved in the signaling transduction among diverse abiotic stress. Under ABA treatment, all six genes showed significantly up-regulated expression. *TaGGCT8*, *TaGGCT11*, *TaGGCT16*, *TaGGCT17*, and *TaGGCT20* were induced after 6 h treatment and reached peak at 12 h, while *TaGGCT13* reached the highest expression level after 48 h treatment (Fig. 5e). This suggested that *TaGGCT* genes family could be highly involved in the regulatory pathways of drought, salt, and ABA stresses.

### Association analysis of *TaGGCT20* gene haplotypes with grain-related traits in wheat

**Fig. 6:**
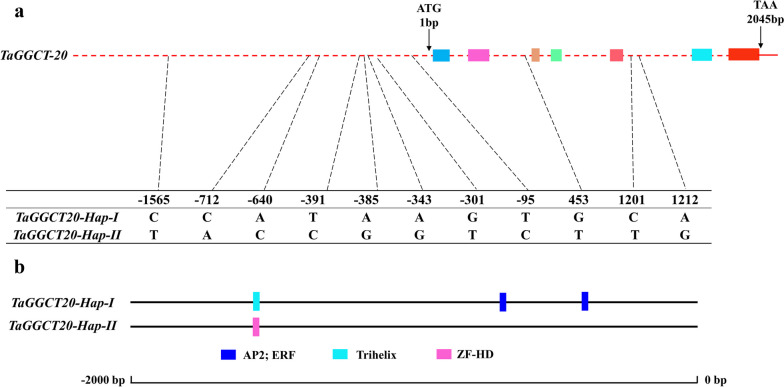
*TaGGCT20* gene structure and main *cis*-element distribution of promoter. **a** *TaGGCT20* gene structure and SNP site of the two haplotypes. **b** Distribution of *cis*-element containing SNP in two haplotype promoters of *TaGGCT20*

**Fig. 7:**
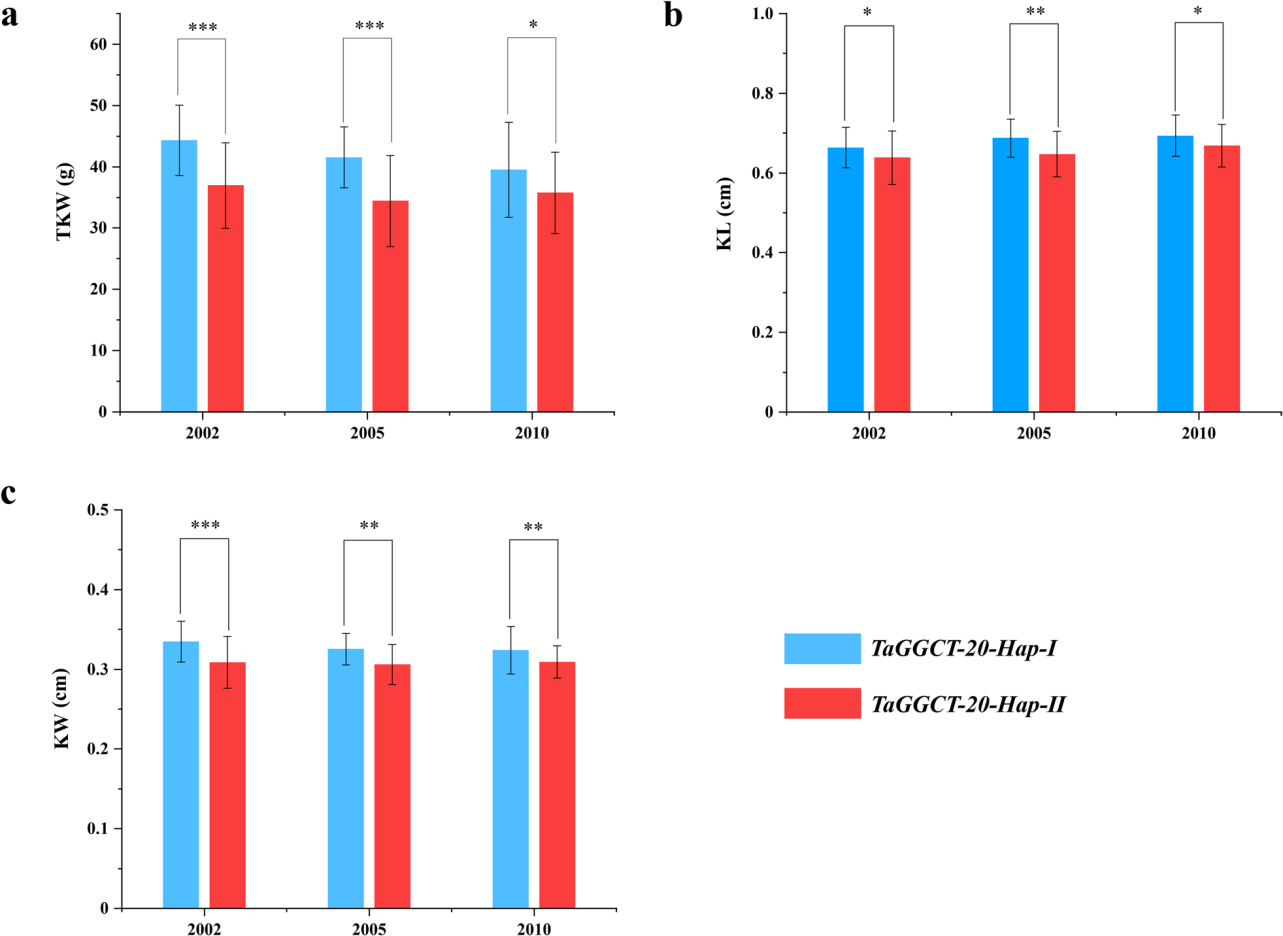
Association analysis of *TaGGCT20* allelic variation and yield traits in different environments. **a** Association analysis of two haplotypes of *TaGGCT20* with TKW in three environments. **b** Association analysis of two haplotypes of *TaGGCT20* with KL in three environments. **c** Association analysis of two haplotypes of *TaGGCT20* with KW in three environments. Note: The X-axis represents different environments (Luoyang, 2002; Luoyang, 2005; Xinxiang, 2006; Shunyi, 2010). The values show the mean ± SD (*n* > 50 seeds). Significant statistical analysis was carried out by Student’s t -test (*, *P*  < 0.05; **, *P*  < 0.01)

Based on the wheat RNA-Seq data and RT-qPCR results, we found that some genes of *TaGGCT* family were highly expressed during wheat grain development. Thus, we further detected polymorphisms in the 2000 bp DNA fragment of the promoter and coding region of *TaGGCT* genes by using genomic sequence data of 677 varieties acquired from the Wheat Union website (http://wheat.cau.edu.cn/WheatUnion/). The results showed that a total of 11 SNPs (single-nucleotide polymorphisms) variants were revealed by the analysis of SNP at promoter and coding regions of the *TaGGCT20* gene. These variable sites were located at 1212(A/G), 1201(C/T), 453(G/T), -95(T/C), -301(G/T), -343(A/G), -385(A/G), -391(T/C), -640(A/C), -712(C/A), and - 1565(C/T) bp positions. Based on these variable sites, two haplotypes were formed and named *TaGGCT20-Hap-I* and *TaGGCT20-Hap-II* (Fig. 6a). Bioinformatic analysis showed that the SNPs located in the promoter of *GGCT* genes may participate in the formation of several transcription factor binding sites, such as ERF and ZF-HD binding site (Fig. 6b). To detect the effect of *TaGGCT20* allele variation on yield-related traits in wheat, we used published data from 122 of these 677 varieties to conduct the associations analysis of *TaGGCT* genes with grain traits (Table S4). We found that the TaG*GCT20* gene with two haplotypes was significantly associated with TKW, KL, and KW in the three environments (*P* < 0.05) (Table S5). The *TaGGCT20-Hap-I* allelic variation had significantly higher TKW, KL and KW, compared to *TaGGCT20-Hap-II* (Fig. 7a, b and c). Therefore, the transcription factor binding site specifically contained in the promoter of *TaGGCT20* gene in *TaGGCT20-Hap-I* might be involved in regulating wheat grain development and increasing yield.

### Selection of *TaGGCT20* haplotypes in wheat breeding process

**Fig. 8:**
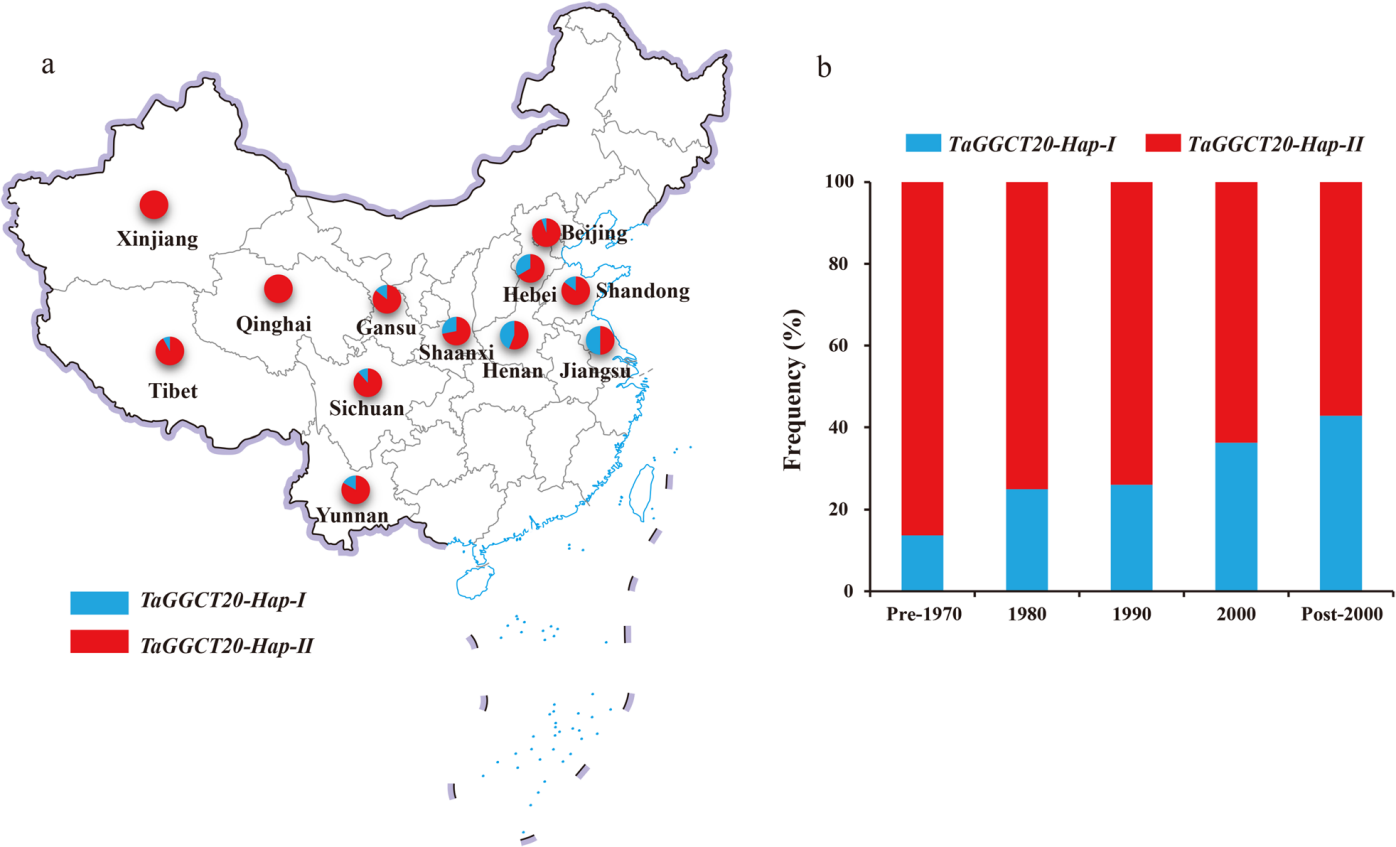
Spatial and temporal distribution of *TaGGCT20* haplotype. **a** Geographic distribution of varieties with *TaGGCT20* haplotypes in China. **b** Frequencies of *TaGGCT20* allelic variation in Chinese wheat breeding programs in different decades

Breeding selection demonstrates the gradual accumulation of superior haplotypes during the breeding process, leaving a deep imprint in the genome. To investigate whether the *TaGGCT20* gene was positively selected in wheat breeding, the geographical distribution of the two haplotypes of the *TaGGCT20* gene was assessed using 370 wheat varieties from 12 growing regions in China (Table S6). Meanwhile, 109 varieties were used to determine the frequency of these two haplotypes in the collection of landraces and modern varieties in China. The results showed that *TaGGCT20-Hap-II* were extensively distributed in the wheat growing regions (Fig. 8a), while the frequency of favorite *TaGGCT20-Hap-I* with high TKW were increased from 13.64% in the 1970s to 42.86% after 2000 (Fig. 8b).

## Discussion

GSH plays a crucial role in plant growth and development, as well as responses to various abiotic stress [26]. As a key antioxidant, GSH is considered as a regulator to maintain cellular redox status in cells, thus indirectly affecting cellular activity [27, 28]. As key enzymes involved in the pathway of glutathione degradation, some GGCT proteins has been identified and functional analyzed in Arabidopsis and rice [2, 29], but *GGCT* genes has not yet been studied in wheat. In this study, 20 *TaGGCT* genes were identified in wheat and their family members were comprehensively analyzed to explore the functions of the *TaGGCT* family genes (Table S1).

Comparisons between genomes of different organisms can provide rapid insights into the genomic structure and their biological function [30]. According to previous findings in Arabidopsis and rice, *GGCT* genes were classified into three different subfamilies (Fig. 1), consistent with our results according to the phylogenetic tree analysis [2, 30]. Previous studies revealed that genes typically undergo tandem repeat events to amplify gene family members during the evolutionary process [31]. Meanwhile, gene duplication can cause functional divergence among genes and expedite the emergence of novel genes [10, 32–35]. In this study, a large amount of gene duplication was identified in *TaGGCT* family with the Ka/Ks ratio of *GGCT* gene pairs less than 1 (Table S2). This indicated that the *TaGGCT* genes might have undergone purification selection pressure and were retained to promote the expansion of *GGCT* genes in wheat. For example, *TaGGCT15*, as a separate branch in phylogenetic tree, missed numbers of conserved motifs compared with other *GGCT* genes and might lead to functional differentiation.

As one of the most important food crops, wheat is affected by diverse environmental stresses, leading to substantial yield loss [36, 37]. Previous studies showed that the *GGCT* genes were involved in plant abiotic stress response, such as drought, salinity, and sulfur starvation [38]. Considering that the promoter regions of *TaGGCT* family members also contained many *cis*-elements related to plant hormones and stress, such as ABRE, DRE, MYB and LTR (Fig. S4, Table S3), we analyzed the expression patterns of *TaGGCT* genes under different abiotic stresses using the RNA-Seq database and validated the results with RT-qPCR. The expression levels of *TaGGCT* genes were differentially regulated in response to drought and salt stress treatments (Fig. 5). Overall, these results suggested that *TaGGCT* genes might be involved in the plant responses to drought stresses.

The *cis*-acting sequence of abscisic acid responsive element (ABRE) has been identified in the promoters of *TaEm*, *Osem* and *Rab16A-D*, as well as barley *HVA22* and *HVA1*, playing a key role in plant response to environmental stress [39]. In this study, RT-qPCR analysis revealed that the expression of *TaGGCT8*, *TaGGCT11*, *TaGGCT16*, *TaGGCT17*, and *TaGGCT20* were up-regulated and peaked at 12 h after ABA treatment, while *TaGGCT13* were reached at the highest level after 48 h (Fig. 5). It is consistent with the fact that the promoter region of *TaGGCT* gene family members contains many abscisic acid response elements (Table S3). This finding suggested that the *TaGGCT* genes could play an important role in the response to drought and salt stress via the ABA-dependent pathway.

Breeding new varieties with high-yield potential and stress tolerance is one of the main challenges for breeders. Long-term breeding selection has greatly affected the current genetic and phenotypic variation in common wheat [40]. Therefore, the application of molecular maker and identify superior haplotypes are effective ways to improve breeding accuracy and efficiency [41]. Many superior haplotypes affecting yield have been identified in wheat, such as *TaSus2-2B*, *TaGS1a* and *TaTPP-6AL1* [42–44]. In this study, we identified two haplotypes of the *TaGGCT20* gene, and the *TaGGCT20-Hap-I* had significantly higher TKW, KL, and KW than *TaGGCT20-Hap-II* (Fig. 7a, b and c). Although *TaGGCT20-Hap-I* was not adequately selected in most regions of China, a significant increase in *TaGGCT20-Hap-I* had occurred in modern varieties in major wheat production areas in recent decades (Fig. 8a and b). The possible reason was the lack of well understanding about the genome information of local wheat varieties in the early breeding process. Therefore, the superior haplotype *TaGGCT20-Hap-I* were gradually positively selected in the historical wheat breeding process.

## Materials and methods

### Plant material and growing conditions

A wheat variety Jinmai 47 was provided by Dryland Wheat Breeding Group of Shanxi Academy of Agricultural Sciences and propagated in the laboratory. The culture conditions of wheat seedlings: 65% humidity, 22–25 °C, light/night, 20,000 lx light intensity, and 16/8-hour cycle. The Hoagland nutrient solution was replaced every five days to ensure a constant supply of nutrients. Seedlings were treated with PEG (20% PEG 6000), NaCl (200 mM) and ABA (100 µM) at three-leaf stage (approximately 12 days). The first leaves of the wheat seedlings were collected at 0, 6, 12, 24 and 48 h after treatment, stored at -80 °C refrigerator. Untreated wheat seedlings were transferred in pots to culturing. Then root, stem and leaf at anthesis, developing spikes, spikes at anthesis, developing grain at 5, 10, 15, and 20 DAA were collected respectively, immediately frozen with liquid nitrogen, and stored at -80 °C until further use.

### Identification of *TaGGCT* genes members in wheat

To identify the *GGCT* genes in wheat, the wheat whole genome data, CDS sequences, protein sequences and annotation files were downloaded from the Ensemble Plants (http://plants.ensembl.org) database [45]. We identified GGCT sequences using Hidden Markov Model (HMM) [46] and Basic Local Alignment Search Tool (BLAST) [47] searches through Chinese Spring reference genome V1.1 (IWGCS RefSeq v1.1). The GGCT conserved structure domain HMMER file (PF04752) as the query sequence was downloaded [48]. The HMMER 3.0 software was used to search the whole genome protein sequence to obtain the wheat *TaGGCT* candidate genes. (threshold *E* < 1e-5). The sequence of *TaGGCT* candidate genes in wheat was analyzed by NCBI-CDD (https://www.ncbi.nlm.nih.gov/Structure/bwrpsb/bwrpsb.cgi), Inter Pro (http://www.ebi.ac.uk/interpro/scan.html) and SMART (http://smart.embl-heidelberg.de/) databases to further validated the wheat TaGGCT protein with a GGCT_like structural domain [23–25, 41]. Based on the previously reported phylogenetic clustering of GGCT in Arabidopsis, the identified wheat *GGCT* genes were renamed as *TaGGCTs* [2]. The sequences of the final identified wheat *GGCT* gene family members were submitted to ExPASY (http://web.expasy.org/protparam/) for Mw and pI prediction. The subcellular localization prediction was performed using Gpos-mPLoc (http://www.csbio.sjtu.edu.cn/bioinf/Gpos-multi/) [48, 49].

### Phylogenetic analysis of the *GGCT* gene family

A total of 12 AtGGCTs, 7 OsGGCTs, and 20 TaGGCTs proteins were selected for phylogenetic analysis (Table S7). The protein sequences of wheat, Arabidopsis and rice *GGCT* genes were compared by multiple sequence alignment by the software MEGA11.0. The phylogenetic tree was constructed using the Neighbor-Joining algorithm (NJ) [50].

### Chromosomal localization, gene duplication and homology analysis of the *TaGGCT* genes in wheat

The chromosomal location of the wheat *GGCT* genes were obtained using the Ensemble Plants database (http://ftp.ebi.ac.uk/ensemblgenomes/pub/release-51/plants/gff3/triticum_aestivum/). The TBtools software was applied for chromosome localization [51]. Syntenic analysis was performed using the One Step MCScanX [52, 53] module of TBtools [51] software. The Simple Ka/Ks Calculator module to calculate the rate of synonymous substitution rate and non-synonymous substitution rate for duplicate gene pairs. Here, if the Ka/Ks ratio = 1 means neutral selection, Ka/Ks < 1 represents pure selection and Ka/Ks > 1 represents the trend evolution accelerator with positive selection [54].

### Gene structure and conserved sequence analysis of wheat *TaGGCTs*

The genome and CDS sequence of wheat *GGCT* genes were downloaded from EnsemblPlants (http://plants.ensembl.org/info/data/ftp/index.html) to analyze the *TaGGCT* genes structure by GSDS (http://gsds.cbi.pku.edu.cn/) online software. The protein conserved motifs were identified using the MEME [55] online program (http://meme.nbcr.net/meme) with the motif number set to 20 and visualized using TBtools software [18].

### Analysis of *cis*-acting elements of wheat *TaGGCTs*

The promoter sequences (2000 bp of sequence upstream from the transcription start site) of *TaGGCTs* genes were obtained from the wheat genomics database. The promoter *cis*-acting elements of *TaGGCTs* genes were predicted by PlantCARE database (http://bioinformatics.psb.ugent.be/webtools/plantcare/) to analyze the *cis*-elements species, number and function [45], and visualized through the TBtools software.

### Analysis of the gene expression pattern of *TaGGCTs* in wheat

The RNA-Seq data of Chinese Spring [56] in different tissues/organs of the wheat variety of Chinese spring at the different development periods were downloaded from the WheatOmics1.0 (http://202.194.139.32/expression/wheat.html) [57] to analyze the differential expression characteristics of *TaGGCT* genes family members. The data was visualized and mapped based on the TBtools software [58].

### RNA extraction and quantitative real-time PCR analyses

Total RNA was extracted from the collected samples using the TIAN GEN.® Plant Tissue RNA Rapid Extraction Kit according to the instructions. First-strand cDNA was synthesized using FastKing gDNA Dispelling RT SuperMix (BEIJING). RT-qPCR analysis was performed using FastReal qPCR PreMix (SYBR Green) to detect changes in the relative expression of six genes in different tissues and under different stress treatments according to the method. Wheat *TaTubulin* [59] was used as reference gene for wheat tissue development expression analysis, and wheat *TaActin1* [60] was used as reference gene for wheat stress expression analysis. The 2^−ΔΔCt^ method was used to calculate relative transcription levels normalized. All quantifications were performed in three biological replicates. The primers used for RT-qPCR were listed in Table S8.

### Association analysis of the *TaGGCT20* gene with seed-related traits in wheat

The 677 hexaploid wheat resequencing data in WheatUnion database (http://wheat.cau.edu.cn/WheatUnion/?language=en) was used to obtain the variant site of the *TaGGCT* genes [18–22]. Using the software SPSS 26, the phenotypic differences between genotypes were determined based on one-way ANOVA. The PlantPAN3.0 (http://plantpan.itps.ncku.edu.tw) was used to predict the *cis*-acting element in the 2000 bp promoter region of *TaGGCT20* gene. A total of 370 strains from the WheatUnion database were used to analyze the geographical distribution of *TaGGCT20* two haplotypes in China, and 109 strains were used to analyze the selection of *TaGGCT20* two haplotypes in the different decades in China. The phenotypic data of 122 wheat test strains were obtained from published article [1, 20, 21]. They determined TKW, KL, KW in Luoyang, Henan Province in 2002 and 2005 and Shunyi, Beijing in 2010. All of material information were listed in Tables S4, S6, S9 and S10.

### Statistical analysis

One-way analysis of variance (ANOVA) was performed using IBM SPSS Statistics 25.0 software (http://www.ibm.com) to determine the significance of different haplotypes with respect to phenotypic traits.

### Supplementary Information

**Additional file 1: Fig. S1.** Conserved domains of *TaGGCTs* in wheat. **Fig. S2.** Distribution of 20 *TaGGCT* genes on wheat chromosomes. **Fig. S3.** Collinearity analysis of *GGCT* genes in rice, wheat and maize. **Fig. S4.** Analysis of *cis*-elements in the promoter of *TaGGCT* genes.

**Additional file 2: Table S1.** Characteristics of *TaGGCT* gene family members. **Table S2.** *TaGGCT* genes duplication events in wheat. **Table S3.** Analysis of *cis*-elements in the promoter of *TaGGCT* genes. **Table S4.** Variation sites and genotypes of *TaGGCT20* gene in 677 wheat materials. **Table S5.** Association analysis of TKW, KL and KW between two haplotypes of *TaGGCT20* gene. **Table S6.** The information of the wheat diversity panel and their genotypes distribution of *TaGGCT20* alleles. **Table S7.**  Members of the *GGCT* genes family in rice and Arabidopsis. **Table S8.**  Primer sequences used in this study. **Table S9.** The information of the wheat diversity panel and their genotypes of *TaGGCT20* alleles in the different decades. **Table S10.** Grain phenotypic traits for association analysis.

## Data Availability

The relevant data and additional information are available in the supplementary files.

## Abbreviations

ABRE: Abscisic acid responsive element
BLAST: Basic Local Alignment Search Tool
DAA: Days after anthesis
GGCT: γ-glutamylcyclotransferase
GSH: Glutathione
HMM: Hidden Markov Model
Ka: Non-synonymous substitution rate
KL: Kernel length
Ks: Synonymous substitution rate
KW: Kernel width
Mw: Molecular weight
NJ: Neighbour-joining
pI: Isoelectric poin
RT-qPCR: Real-time quantitative polymerase chain reaction
SNP: Single nucleotide polymorphism
SURE: SUlfur response element
TKW: Thousand-kernel weight
5OP: 5-oxoproline

## References

[1] Yang Z, Wang Z, Wang W, Xie X, Chai L, Wang X, Feng X, Li J, Peng H, Su Z (2022). ggComp enables dissection of germplasm resources and construction of a multiscale germplasm network in wheat. Plant Physiol.

[2] Kumar S, Kaur A, Chattopadhyay B, Bachhawat Anand K (2015). Defining the cytosolic pathway of glutathione degradation in *Arabidopsis thaliana*: role of the ChaC/GCG family of γ-glutamyl cyclotransferases as glutathione-degrading enzymes and AtLAP1 as the cys-gly peptidase. Biochem J.

[3] Noctor G, Mhamdi A, Chaouch S, Han Y, Neukermans J, Marquez-Garcia B, Queval G, Foyer CH (2012). Glutathione in plants: an integrated overview. Plant Cell Environ.

[4] Noctor G, Foyer CH (1998). Ascorbate and glutathione: keeping active oxygen under control. Annu Rev Plant Physiol Plant Mol Biol.

[5] Noctor G, Queval G, Mhamdi A, Chaouch S, Foyer CH (2011). Glutathione. Arabidopsis Book.

[6] Paulose B, Chhikara S, Coomey J, Jung HI, Vatamaniuk O, Dhankher OP (2013). A γ-glutamyl cyclotransferase protects arabidopsis plants from heavy metal toxicity by recycling glutamate to maintain glutathione homeostasis. Plant Cell.

[7] Grzam A, Martin MN, Hell R, Meyer AJ (2007). γ-Glutamyl transpeptidase GGT4 initiates vacuolar degradation of glutathiones-conjugates in Arabidopsis. FEBS Lett.

[8] Kovalchuk I, Titov V, Hohn B, Kovalchuk O (2005). Transcriptome profiling reveals similarities and differences in plant responses to cadmium and lead. Mutat Res.

[9] Gong Q, Li P, Ma S, Indu Rupassara S, Bohnert HJ (2005). Salinity stress adaptation competence in the extremophile thellungiella halophila in comparison with its relative *Arabidopsis thaliana*. Plant J.

[10] Maruyama-Nakashita A, Nakamura Y, Watanabe-Takahashi A, Inoue E, Yamaya T, Takahashi H (2005). Identification of a novel *cis*-acting element conferring sulfur deficiency response in arabidopsis roots. Plant J.

[11] Hubberten H-M, Drozd A, Tran BV, Hesse H, Hoefgen R (2012). Local and systemic regulation of sulfur homeostasis in roots of *Arabidopsis thaliana*. Plant J.

[12] Bielecka M, Watanabe M, Morcuende R, Scheible W-R, Hawkesford MJ, Hesse H, Hoefgen R (2015). Transcriptome and metabolome analysis of plant sulfate Starvation and resupply provides novel information on transcriptional regulation of metabolism associated with sulfur, nitrogen and phosphorus nutritional responses in arabidopsis. Front Plant Sci.

[13] Joshi NC, Meyer AJ, Bangash SAK, Zheng ZL, Leustek T (2018). Arabidopsis γ-glutamylcyclotransferase affects glutathione content and root system architecture during sulfur starvation. New Phytol.

[14] Ghosh A, Islam MS, Alam NB, Mustafiz A, Islam T (2022). Transcript profiling of glutathione metabolizing genes reveals abiotic stress and glutathione-specific alteration in Arabidopsis and rice. Physiol Mol Biology Plants.

[15] Appels R, Eversole K, Stein N, Feuillet C, Keller B, Rogers J, Pozniak CJ, Choulet F, Distelfeld A, Poland J (2018). Shifting the limits in wheat research and breeding using a fully annotated reference genome. Science.

[16] Marcussen T, Sandve SR, Heier L, Spannagl M, Pfeifer M, Jakobsen KS, Wulff BBH, Steuernagel B, Mayer KFX, Olsen O-A (2014). Ancient hybridizations among the ancestral genomes of bread wheat. Science.

[17] Pasternak M, Lim B, Wirtz M, Hell R, Cobbett CS, Meyer AJ (2008). Restricting glutathione biosynthesis to the cytosol is sufficient for normal plant development. Plant J.

[18] Zhou Y, Zhao X, Li Y, Xu J, Bi A, Kang L, Xu D, Chen H, Wang Y, Wang Y (2020). Triticum population sequencing provides insights into wheat adaptation. Nat Genet.

[19] Brinton J, Uauy C (2019). A reductionist approach to dissecting grain weight and yield in wheat. J Integr Plant Biol.

[20] Cheng H, Liu J, Wen J, Nie X, Xu L, Chen N, Li Z, Wang Q, Zheng Z, Li M (2019). Frequent intra- and inter-species introgression shapes the landscape of genetic variation in bread wheat. Genome Biol.

[21] Guo W, Sun Q, Yao Y, Peng H, Xin M, Hu Z, Ni Z, Li X, Wang Z, Wang W (2020). SnpHub: an easy-to-set-up web server framework for exploring large-scale genomic variation data in the post-genomic era with applications in wheat. Gigascience.

[22] Hao C, Jiao C, Hou J, Li T, Liu H, Wang Y, Zheng J, Liu H, Bi Z, Xu F (2020). Resequencing of 145 Landmark cultivars reveals asymmetric sub-genome selection and strong founder genotype effects on wheat breeding in China. Mol Plant.

[23] El-Gebali S, Mistry J, Bateman A, Eddy SR, Luciani A, Potter SC, Qureshi M, Richardson LJ, Salazar GA, Smart A (2019). The pfam protein families database in 2019. Nucleic Acids Res.

[24] Letunic I, Khedkar S, Bork P (2021). SMART: recent updates, new developments and status in 2020. Nucleic Acids Res.

[25] Yang M, Derbyshire MK, Yamashita RA, Marchler-Bauer A (2019). NCBI’s conserved domain database and tools for protein domain analysis. Curr Protoc Bioinformatics.

[26] Ball L, Accotto G-P, Bechtold U, Creissen G, Funck D, Jimenez A, Kular B, Leyland N, Mejia-Carranza J, Reynolds H (2004). Evidence for a direct link between glutathione biosynthesis and stress defense gene expression in arabidopsis. Plant Cell.

[27] Schafer FQ, Buettner GR (2001). Redox environment of the cell as viewed through the redox state of the glutathione disulfide/glutathione couple. Free Radic Biol Med.

[28] Cooper CE, Patel RP, Brookes PS, Darley-Usmar VM (2002). Nanotransducers in cellular redox signaling: modification of thiols by reactive oxygen and nitrogen species. Trends Biochem Sci.

[29] Chang M, Ma J, Sun Y, Tian L, Liu L, Chen Q, Zhang Z, Wan X, Sun J (2023). γ-Glutamyl‐transpeptidase CsGGT2 functions as light‐activated theanine hydrolase in tea plant (*Camellia sinensis* L). Plant Cell Environ.

[30] Yang Y, Xu T, Wang H, Feng D (2021). Genome-wide identification and expression analysis of the TaYUCCA gene family in wheat. Mol Biol Rep.

[31] Cannon SB, Mitra A, Baumgarten A, Young ND, May G (2004). The roles of segmental and tandem gene duplication in the evolution of large gene families in *Arabidopsis thaliana*. BMC Plant Biol.

[32] Errum A, Rehman N, Khan MR, Ali GM (2021). Genome-wide characterization and expression analysis of pseudo-response regulator gene family in wheat. Mol Biol Rep.

[33] Yu K, Feng M, Yang G, Sun L, Qin Z, Cao J, Wen J, Li H, Zhou Y, Chen X (2020). Changes in alternative splicing in response to domestication and polyploidization in wheat. Plant Physiol.

[34] Su J, Song S, Wang Y, Zeng Y, Dong T, Ge X, Duan H (2023). Genome-wide identification and expression analysis of DREB family genes in cotton. BMC Plant Biol.

[35] Song C, Cao Y, Dai J, Li G, Manzoor MA, Chen C, Deng H (2022). The multifaceted roles of MYC2 in plants: toward transcriptional reprogramming and stress tolerance by Jasmonate Signaling. Front Plant Sci.

[36] Sun A, Li Y, He Y, Zou X, Chen F, Ji R, You C, Yu K, Li Y, Xiao W (2022). Comprehensive genome-wide identification, characterization, and expression analysis of CCHC-type zinc finger gene family in wheat (*Triticum aestivum* L). Front Plant Sci.

[37] Zhang L, Song Z, Li F, Li X, Ji H, Yang S (2019). RETRACTED ARTICLE: the specific MYB binding sites bound by TaMYB in the GAPCp2/3 promoters are involved in the drought stress response in wheat. BMC Plant Biol.

[38] Joshi NC, Meyer AJ, Bangash SAK, Zheng ZL, Leustek T (2019). Arabidopsis γ-glutamylcyclotransferase affects glutathione content and root system architecture during sulfur Starvation. New Phytol.

[39] Roychoudhury A, Sengupta DNJBP (2009). The promoter-elements of some abiotic stress-inducible genes from cereals interact with a nuclear protein from Tobacco. Biol Plant.

[40] Khan N, Zhang Y, Wang J, Li Y, Chen X, Yang L, Zhang J, Li C, Li L, Ur Rehman S (2022). TaGSNE, a WRKY transcription factor, overcomes the trade-off between grain size and grain number in common wheat and is associated with root development. J Exp Bot.

[41] Pasternak M, Lim B, Wirtz M, Hell R, Cobbett CS, Meyer AJ (2007). Restricting glutathione biosynthesis to the cytosol is sufficient for normal plant development. Plant J.

[42] Jiang Q, Hou J, Hao C, Wang L, Ge H, Dong Y, Zhang X (2010). The wheat (T. Aestivum) sucrose synthase 2 gene (TaSus2) active in endosperm development is associated with yield traits. Funct Integr Genom.

[43] Guo Y, Sun J, Zhang G, Wang Y, Kong F, Zhao Y, Li S (2013). Haplotype, molecular marker and phenotype effects associated with mineral nutrient and grain size traits of TaGS1a in wheat. Field Crop Res.

[44] Zhang P, He Z, Tian X, Gao F, Xu D, Liu J, Wen W, Fu L, Li G, Sui X (2017). Cloning of TaTPP-6AL1 associated with grain weight in bread wheat and development of functional marker. Mol Breeding.

[45] Zhang P, Zhang L, Chen T, Jing F, Liu Y, Ma J, Tian T, Yang D (2022). Genome-wide identification and expression analysis of the *GSK* gene family in wheat (*Triticum aestivum* L). Mol Biol Rep.

[46] Eddy SR (2011). Accelerated Profile HMM searches. PLoS Comput Biol.

[47] Camacho C, Boratyn GM, Joukov V, Vera Alvarez R, Madden TL (2023). ElasticBLAST: accelerating sequence search via cloud computing. BMC Bioinformatics.

[48] Finn RD (2006). Pfam: clans, web tools and services. Nucleic Acids Res.

[49] Newbigin E, Chou K-C, Shen H-B (2010). Plant-mPLoc: a top-down strategy to augment the power for predicting plant protein subcellular localization. PLoS ONE.

[50] Tamura K, Stecher G, Kumar S, Battistuzzi FU (2021). MEGA11: molecular evolutionary genetics analysis version 11. Mol Biol Evol.

[51] Chen C, Chen H, Zhang Y, Thomas HR, Frank MH, He Y, Xia R (2020). TBtools: an integrative toolkit developed for interactive analyses of big biological data. Mol Plant.

[52] Lai W, Zhou Y, Pan R, Liao L, He J, Liu H, Yang Y, Liu S (2020). Identification and expression analysis of stress-associated proteins (SAPs) containing A20/AN1 zinc finger in Cucumber. Plants.

[53] Wang Y, Li J, Paterson AH (2013). MCScanX-transposed: detecting transposed gene duplications based on multiple colinearity scans. Bioinformatics.

[54] Zhang Z, Li J, Zhao X-Q, Wang J, Wong GK-S, Yu J (2006). KaKs_calculator: calculating Ka and Ks through Model selection and model averaging. Genom Proteom Bioinform.

[55] Bailey TL, Boden M, Buske FA, Frith M, Grant CE, Clementi L, Ren J, Li WW, Noble WS (2009). MEME SUITE: tools for motif discovery and searching. Nucleic Acids Res.

[56] Oleson AE, Sasakuma M (1980). Biophysics. S1 nuclease of aspergillus oryzae: a glycoprotein with an associated nucleotidase activity. Archives of biochemistry and biophysics.

[57] Borrill P, Ramirez-Gonzalez R, Uauy C (2016). expVIP: a customizable RNA-seq data analysis and visualization platform. Plant Physiol.

[58] An X, Zhao S, Luo X, Chen C, Liu T, Li W, Zou L, Sun C (2023). Genome-wide identification and expression analysis of the regulator of chromosome condensation 1 gene family in wheat (*Triticum aestivum* L). Front Plant Sci.

[59] Mei F, Chen B, Du L, Li S, Zhu D, Chen N, Zhang Y, Li F, Wang Z, Cheng X (2022). A gain-of-function allele of a DREB transcription factor gene ameliorates drought tolerance in wheat. Plant Cell.

[60] He J, Li C, Hu N, Zhu Y, He Z, Sun Y, Wang Z, Wang Y (2022). ECERIFERUM1-6A is required for the synthesis of cuticular wax alkanes and promotes drought tolerance in wheat. Plant Physiol.

