# Supplementary material for: Genome-wide identification and analysis of the GGCT gene family in wheat
Source: BMC Genomics. 2024 Jan 4;25:32. doi: 10.1186/s12864-023-09934-w (PMC10768367; doi:10.1186/s12864-023-09934-w)
Supplement: Supplementary file 1 — Additional file 1: Fig. S1. Conserved domains of TaGGCTs in wheat. Fig. S2. Distribution of 20 TaGGCT genes on wheat chromosomes. Fig. S3. Collinearity analysis of GGCT genes in rice, wheat and maize. Fig. S4. Analysis of cis-elements in the promoter of TaGGCT genes. [file 12864_2023_9934_MOESM1_ESM.docx]

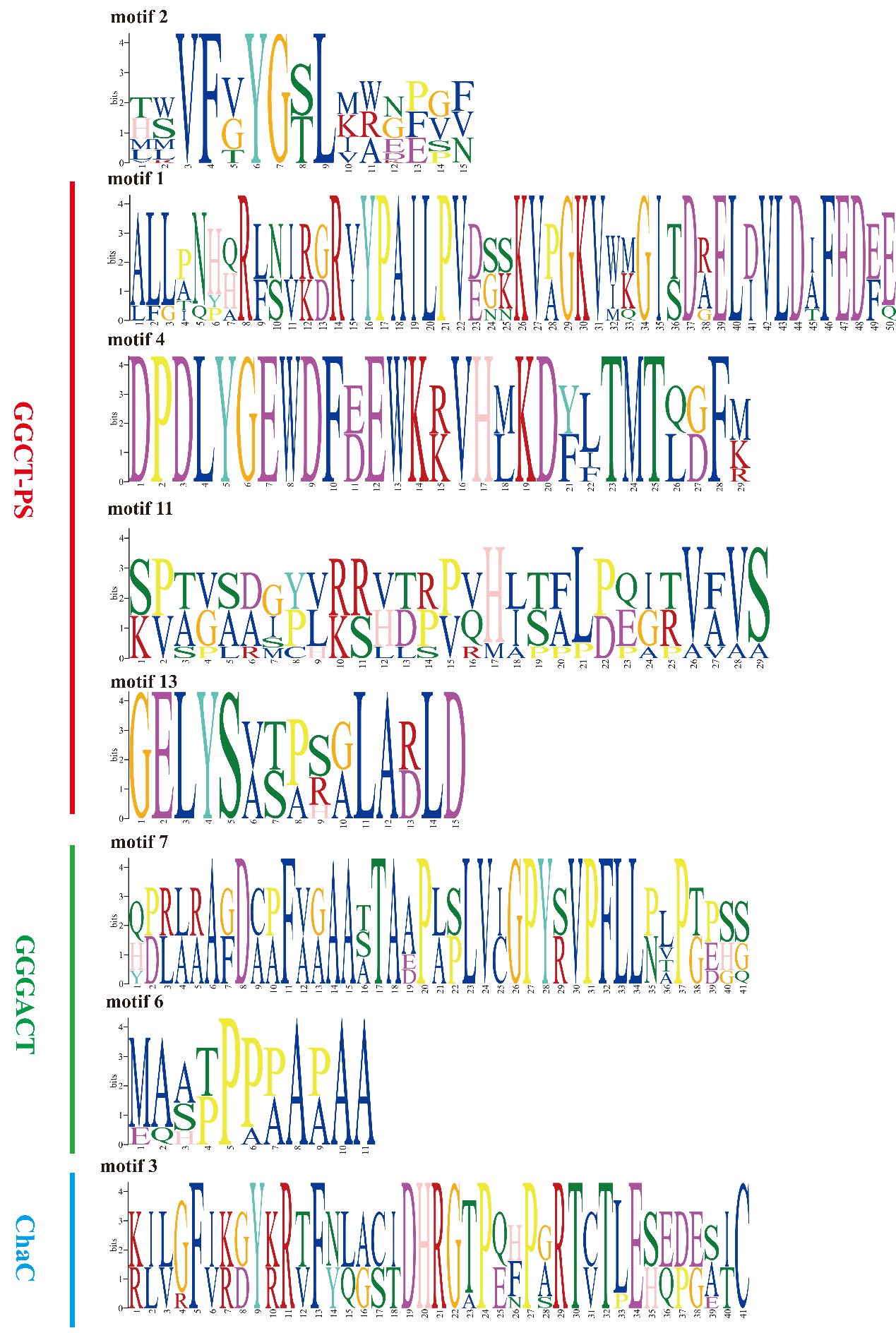


**Fig. S1.** Conserved domains of *TaGGCTs* in wheat.


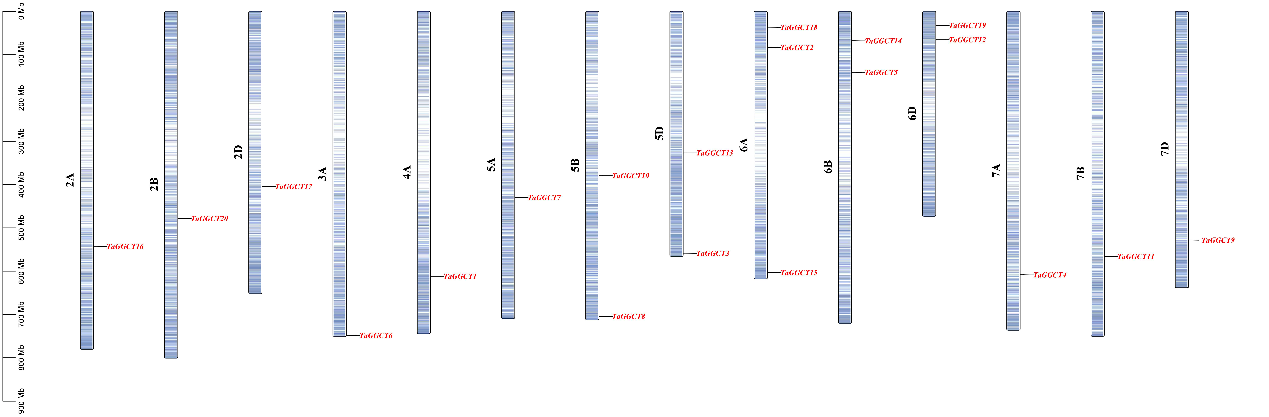


**Fig. S2.** Distribution of 20 *TaGGCT* genes on wheat chromosomes.


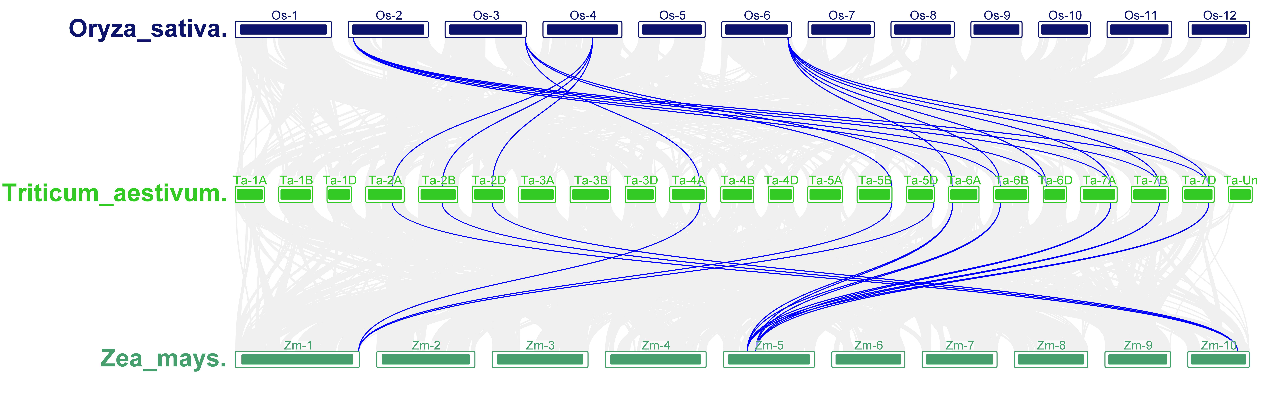


**Fig. S3.** Collinearity analysis of *GGCT* genes in rice, wheat and maize.


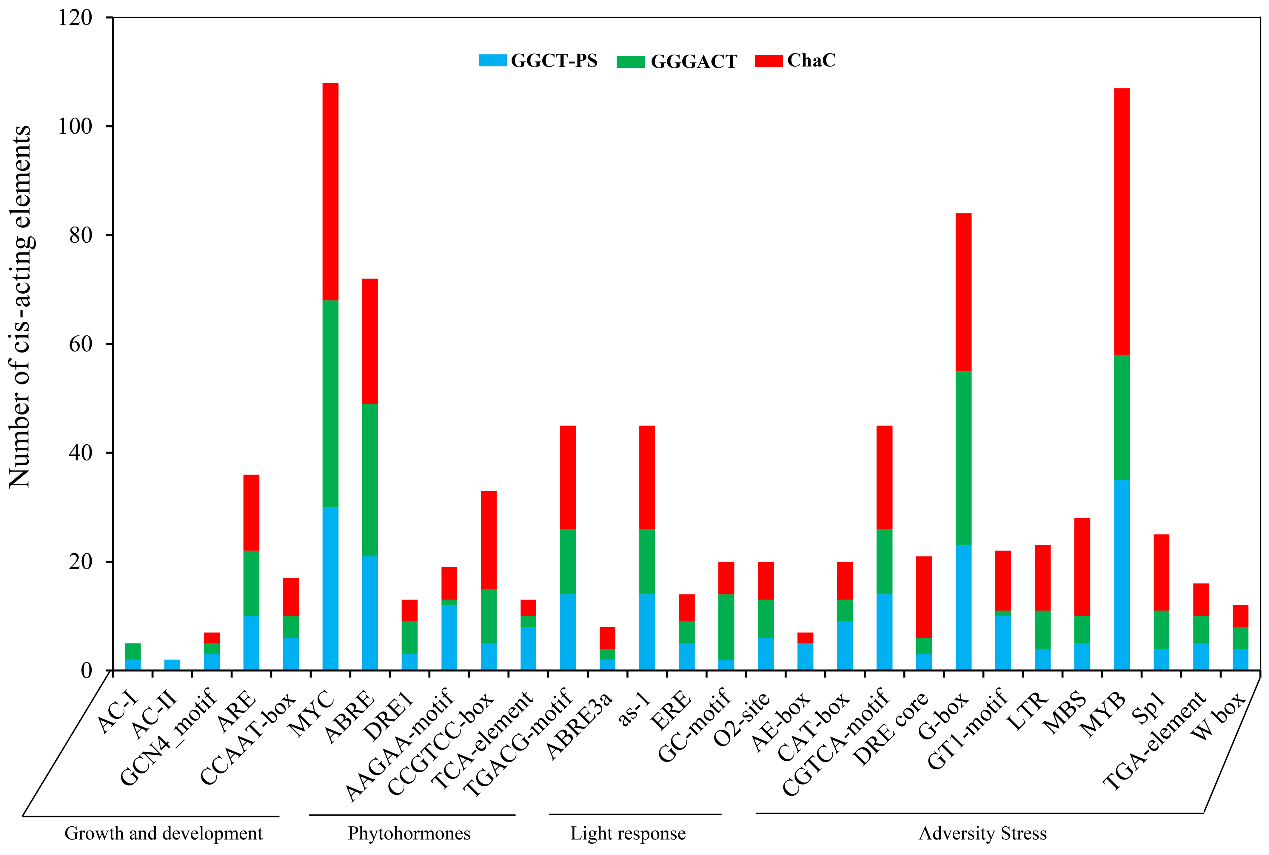


**Fig. S4.** Analysis of *cis*-elements in in the promoter of *TaGGCT* genes.
